# Supplementary material for: Discovery of an inhibitor of DNA-driven inflammation that preferentially targets the AIM2 inflammasome
Source: iScience. 2023 Apr 27;26(5):106758. doi: 10.1016/j.isci.2023.106758 (PMC10193008; doi:10.1016/j.isci.2023.106758)
Supplement: Docuement S1. Figures S1–S10 and Tables S1–S4 [file mmc1.pdf]

iScience, Volume *26*

Supplemental information

Discovery of an inhibitor of DNA-driven inflammation that preferentially targets the AIM2 inflammasome

Jack P. Green, Lina Y. El-Sharkawy, Stefan Roth, Jie Zhu, Jiayu Cao, Andrew G. Leach, Arthur Liesz, Sally Freeman, and David Brough

Supplementary Figure 1

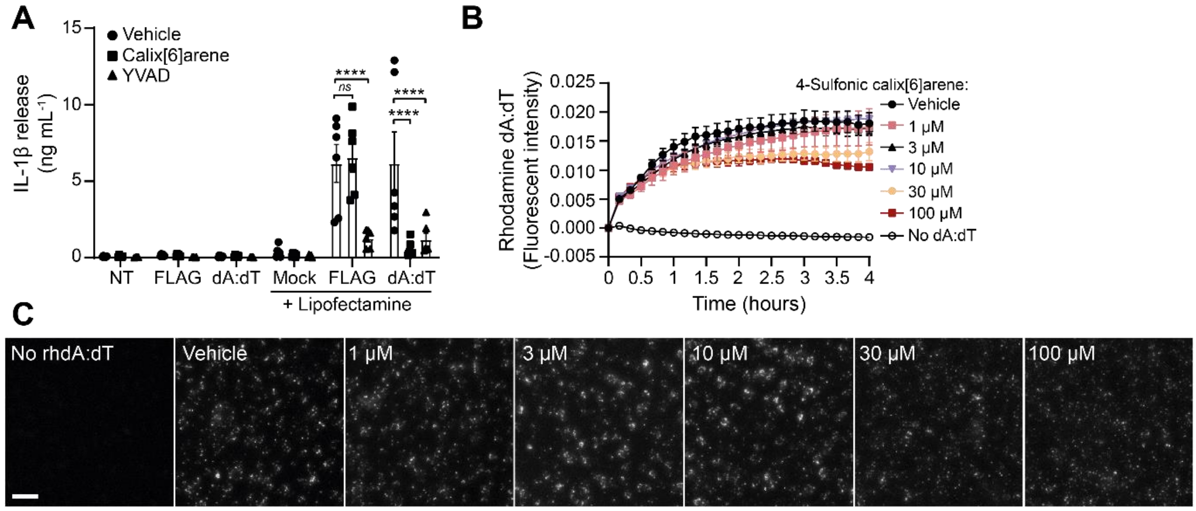

Supplementary Figure 1 – 4-Sulfonic calix[6]arene does not impact transfection. Related to Figure 1. **(A)** IL-1β release in the supernatants of LPS-primed (1 μg mL^{-1}, 4 h) BMDMs. BMDMs were pre-treated with 4-sulfonic calix[6]arene (30 μM), the caspase-1 inhibitor Ac-YVAD-CMK (YVAD, 100 μM) or vehicle control (DMSO) before stimulation with poly dA:dT (1 μg mL^{-1}) or flagellin (1 μg mL^{-1}) in the presence or absence of lipofectamine 3000 for 4 h (n=6). **(B)** Fluorescence intensity of rhodamine-tagged poly dA:dT (1 μg mL^{-1}) transfected into bone marrow-derived macrophages (BMDMs) in the presence of the indicated concentration of 4-sulfonic calix[6]arene (1-100 μM) (n=3). **(C)** Representative images of BMDMs from the experiment in (A) (scale bar = 100 μm). ****p>0.0001 determined by a two-way ANOVA with Dunnett's post hoc analysis vs vehicle control. Values shown are mean ± the SEM.

Supplementary Figure 2

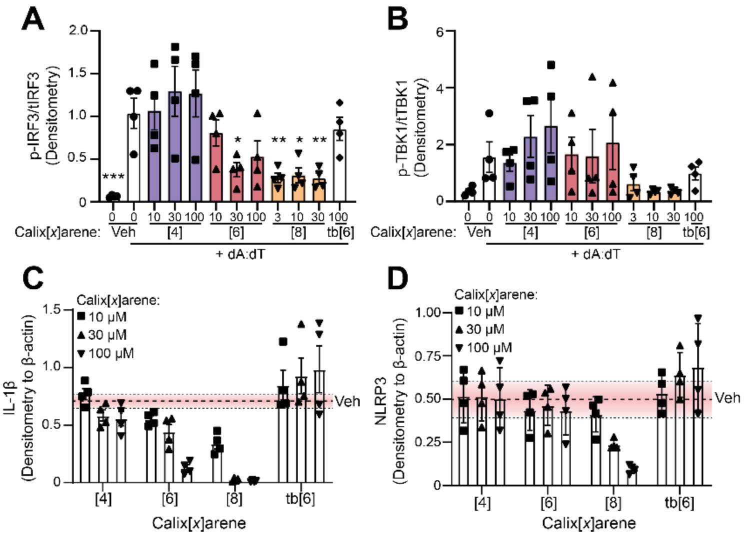

Supplementary Figure 2 – Densitometry from Figure 3: Inhibition of dsDNA inflammatory signalling by 4-sulfonic calixarenes is readily reversible and is mediated by the exposed sulfonic acid groups. Related to Figure 3. **(A)** The ratio of p-IRF3 (Ser396) to total IRF3 and **(B)** the ratio of p-TBK1 to total TBK1 determined by densitometry of experiments shown in Fig 3G (n=4). **(C)** Densitometry of IL-1β and **(D)** NLRP3 from experiments shown in Fig 3I (n=4). *p<0.05, **p<0.01, ***p>0.001 determined by a one-way ANOVA with Dunnett's post hoc analysis vs vehicle control. Values shown are mean ± the SEM.

Supplementary Figure 3

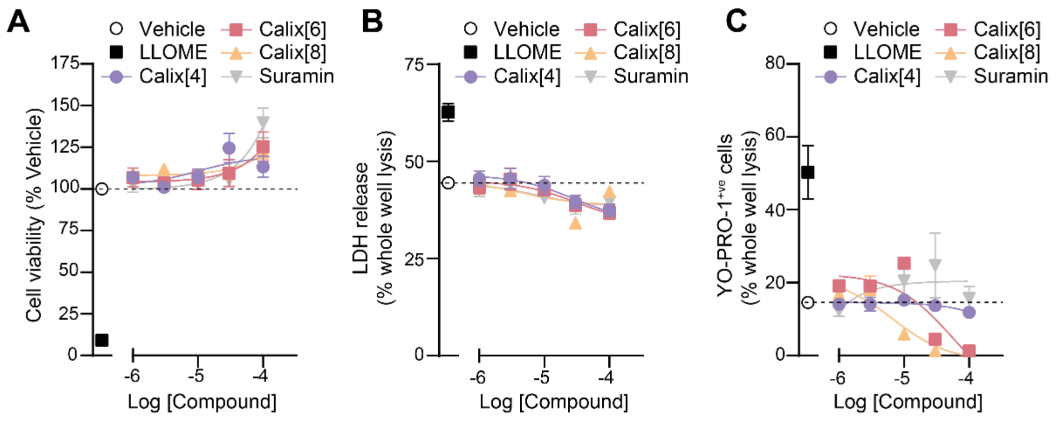

Supplementary Figure 3 – 4-Sulfonic calixarenes and suramin are not cytotoxic in BMDMs. Related to Figure 3. BMDMs were incubated for 24 h with the indicated concentration (1-100 µM) of 4-sulfonic calix[4]arene (Calix[4]), 4-sulfonic calix[6]arene (Calix[6]), 4-sulfonic calix[8]arene (Calix[8]), suramin, or a vehicle control (DMSO (0.5% v/v). LLOME (1 mM, 1 h) was used alongside as a positive control of cell death. **(A)** MTT assay (n=4). **(B)** LDH release from BMDMs into the supernatant (n=3). **(C)** YO-PRO-1^{+ve} cells assessed by microscopy (n=3). Representative images can be found in Supplementary figure 4. Concentration-response curves were fitted using a three parameter logistical (3PL) model. Values shown are mean ± the SEM.

Supplementary Figure 4

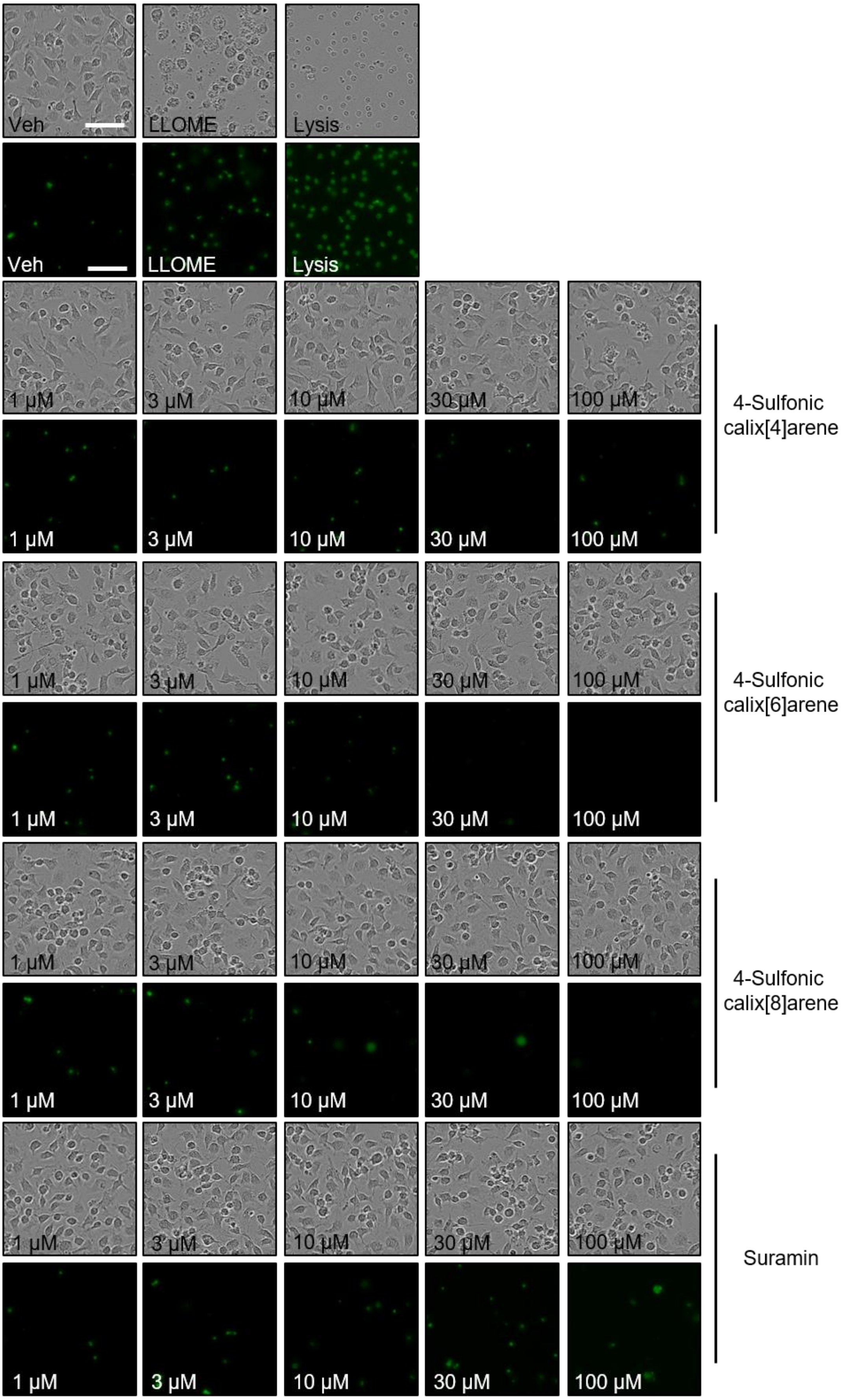

Supplementary Figure 4 - 4-Sulfonic calixarenes and suramin are not cytotoxic in BMDMs. Related to Figure 3. Representative phase contrast and YO-PRO-1 staining in BMDMs treated with the indicated concentration (1-100 µM) 4-sulfonic calix[4]arene, 4-sulfonic calix[6]arene, 4-sulfonic calix[8[arene, suramin or a vehicle control (DMSO, 0.5% v/v) for 24 hr (n=3) (scale bar = 100 µm). LLOME (1 mM, 1 h) and a full lysis (triton x100, 0.25% v/v) was used alongside as a positive control of cell death. Quantification of YO-PRO-1 fluorescence can be found in supplementary figure 3.

Supplementary Figure 5

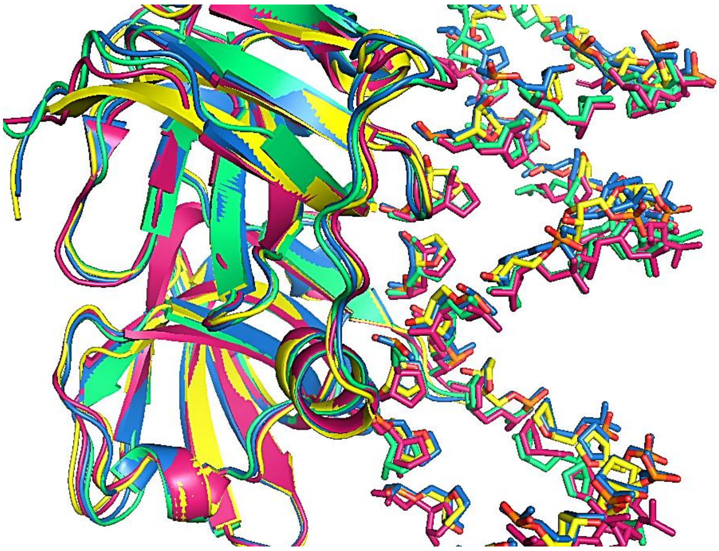

Supplementary Figure 5 - Superimposition of the 4 HIN domains of AIM2 with dsDNA. Related to Figure 4. Phosphate and ribose backbone of dsDNA from the crystal structure bound to each HIN domain showing binding of phosphates in discrete regions, blue: (HIN-a), green (HIN-b), yellow (HIN-c), red (HIN-d) (PDB: 3RN5). Image created using Pymol.

Supplementary figure 6

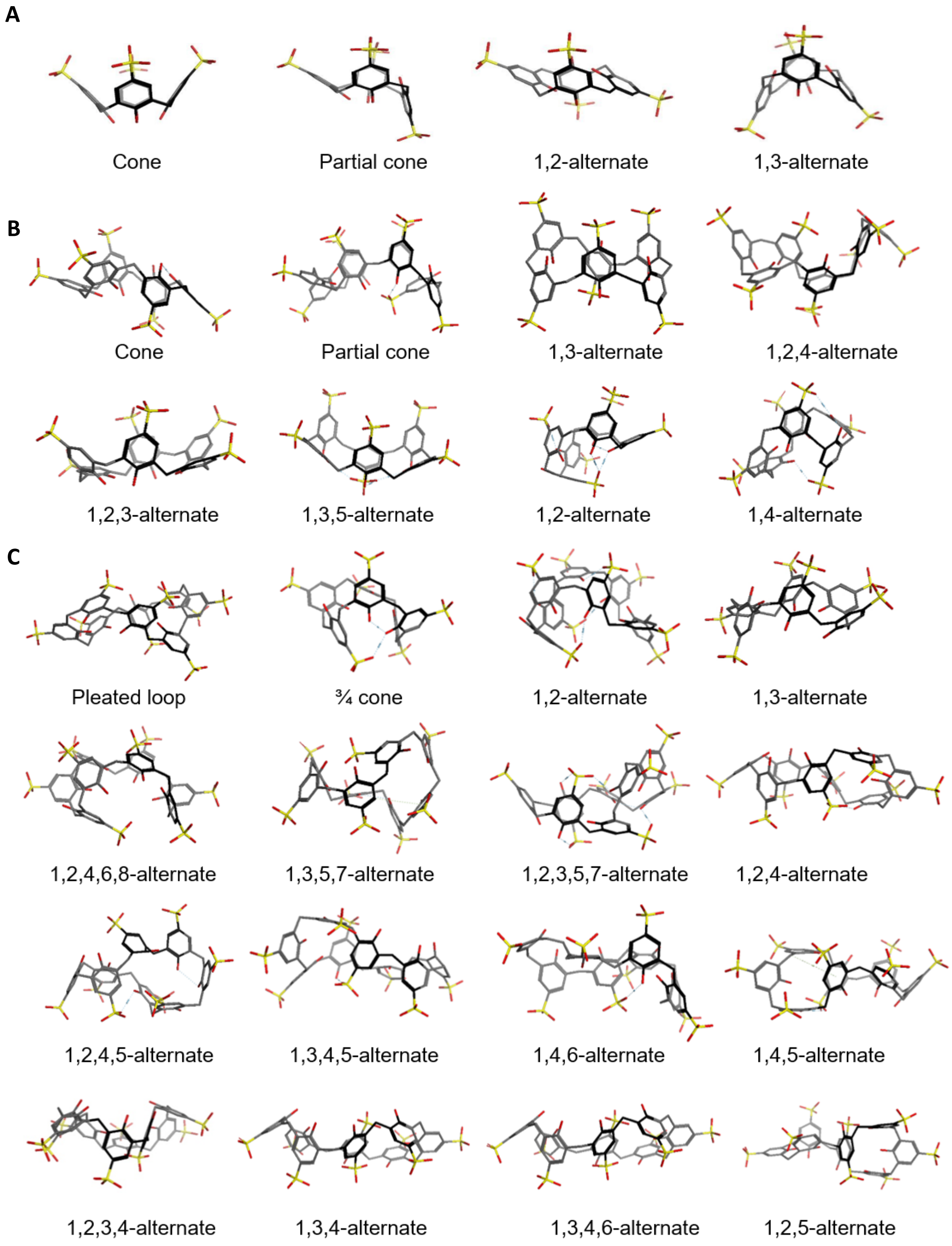

Supplementary Figure 6 - Conformers of 4-sulfonic calixarenes. Related to Figure 4. Stick diagrams for the different conformers of **(A)** 4-sulfonic calix[4]arene, **(B)** 4-sulfonic calix[6]arene and **(C)** 4-sulfonic calix[8]arene using MOE.

Supplementary Table 1 - Top ranked 4-sulfonic calixarene conformers and their energies obtained from a stochastic search in MOE. Related to Figure 4.

	4-Sulfonic calix[4]arene			4-Sulfonic calix[6]arene			4-Sulfonic calix[8]arene		
**#**	**Conformation**	**E (Kcal/mol)**	**ΔE (Kcal/mol)**	**Conformation**	**E (Kcal/mol)**	**ΔE (Kcal/mol)**	**Conformation**	**E (Kcal/mol)**	**ΔE (Kcal/mol)**
**1**	partial cone	238.50	0.00	1,2,4-alternate	335.28	0.00	**1,2,4,6,8-alternate**	431.59	0.00
**2**	cone	239.62	+1.12	1,3,5-alternate	335.30	+0.02	1,2,4,6,8-alternate	433.82	+2.23
**3**	1,3-alternate	241.41	+2.91	1,4-alternate	335.33	+0.05	1,2-alternate	436.07	+4.48
**4**	**1,2-alternate**	241.97	+3.47	1,3,5-alternate	335.36	+0.08	1,2,4,6,8- alternate	437.01	+5.42
**5**	1,2-alternate	241.97	+3.47	1,3,5-alternate	335.39	+0.11	1,2,4,6,8- alternate	437.49	+5.90
**6**	1.2-alternate	241.98	+3.48	1,3,5-alternate	335.44	+0.16	¾ cone	437.58	+5.99
**7**	1,2-alternate	241.99	+3.49	1,3,5-alternate	338.73	+3.45	1,3,5,7-alternate	438.14	+6.55
**8**	1,3-alternate	242.28	+3.78	1,3,5-alternate	338.77	+3.49	¾ cone	438.17	+6.58
**9**	1,3-alternate	242.28	+3.78	1,3,5-alternate	338.86	+3.58	¾ cone	438.37	+6.78
**10**	1,2-alternate	242.32	+3.82	1,2,4-alternate	338.86	+3.58	1,2,4,6,8- alternate	438.66	+7.07
**11**	1,3-alternate	242.35	+3.85	1,2,4-alternate	338.87	+3.59	1,3,5,7-alternate	439.17	+7.58
**12**	1,3-alternate	242.36	+3.86	1,2,4-alternate	338.90	+3.62	1,2,4,6,8- alternate	440.26	+8.67
**13**	1,3-alternate	242.37	+3.87	1,4-alternate	338.92	+3.64	1,3,5,7-alternate	440.44	+8.85
**14**	1,3-alternate	242.73	+4.23	1,3,5-alternate	338.93	+3.65	1,2,4,6,8- alternate	440.77	+9.18
**15**	partial cone	242.78	+4.28	1,3,5-alternate	338.94	+3.66	1,2,4,6,8- alternate	440.80	+9.21
**16**	partial cone	242.78	+4.28	1,3,5-alternate	338.94	+3.66	1,3,5,7-alternate	440.81	+9.22
**17**	1,3-alternate	242.78	+4.28	1,3,5-alternate	339.00	+3.72	1,3,5,7-alternate	440.82	+9.23
**18**	1,3-alternate	242.80	+4.3	1,3,5-alternate	339.00	+3.72	1,2,3,4-alternate	440.88	+9.29
**19**	1,3-alternate	242.81	+4.31	1,3,5-alternate	339.00	+3.72	1,2,4,6,8- alternate	440.89	+9.30
**20**	1,3-alternate	242.86	+4.36	1,3,5-alternate	339.01	+3.73	1,3,5,7-alternate	440.89	+9.30
**21**	1,2-alternate	242.88	+4.38	1,3,5-alternate	339.06	+3.78	1,3,5,7-alternate	441.11	+9.52
**22**	1,3-alternate	242.88	+4.38	1,3,5-alternate	339.07	+3.79	1,2,4,6,8- alternate	441.41	+9.82
**23**	partial cone	242.89	+4.39	1,3,5-alternate	339.09	+3.81	1,3,5,7-alternate	441.422	+9.832
**24**	partial cone	242.90	+4.40	1,3,5-alternate	339.12	+3.84	1,3,5,7-alternate	442.09	+10.50
**25**	partial cone	242.90	+4.40	1,2,4-alternate	339.15	+3.87	1,2,4,5-alternate	442.16	+10.57
**26**	partial cone	242.90	+4.40	1,2,4-alternate	339.16	+3.88	1,2,4,6,8- alternate	442.50	+10.91
**27**	partial cone	242.90	+4.40	1,2,4-alternate	339.16	+3.88	1,2,4,6,8- alternate	442.57	+10.98
**28**	1,3-alternate	242.91	+4.41	1,2,4-alternate	339.16	+3.88	1,2,4,6,8- alternate	442.58	+10.99
**29**	partial cone	243.26	+4.76	1,2,4-alternate	339.18	+3.90	1,2,4,6,8- alternate	442.64	+11.05
**30**	1,3-alternate	243.33	+4.83	1,2,4-alternate	339.23	+3.95	1,2,4,6,8- alternate	442.92	+11.33
**31**	partial cone	243.34	+4.84	1,3,5-alternate	339.33	+4.05	Pleated loop	443.15	+11.56
**32**	1,3-alternate	243.77	+5.27	1,3,5-alternate	339.33	+4.05	1,2,4,6,8- alternate	443.17	+11.58
**33**	cone	243.80	+5.30	1,2,4-alternate	339.35	+4.07	1,2,4,6,8- alternate	443.20	+11.61
**34**	1,3-alternate	243.80	+5.30	1,3,5-alternate	339.39	+4.11	1,2,3,5,7- alternate	443.21	+11.62
**35**	1,3-alternate	243.85	+5.35	1,3,5-alternate	339.41	+4.13	1,2,3,5,7- alternate	443.21	+11.62
**36**	1,3-alternate	243.96	+5.46	1,3,5-alternate	339.41	+4.13	1,2,3,5,7- alternate	443.26	+11.67
**37**	partial cone	243.98	+5.48	1,3,5-alternate	339.45	+4.17	1,2,4,6,8- alternate	443.33	+11.74
**38**	1,3-alternate	243.98	+5.48	1,3,5-alternate	339.61	+4.33	Pleated loop	443.41	+11.82
**39**	partial cone	244.24	+5.74	1,3,5-alternate	339.61	+4.33	1,3,5,7-alternate	443.45	+11.86
**40**	1,3-alternate	244.26	+5.76	1,3,5-alternate	339.76	+4.48	1,2,4,6,8- alternate	433.58	+1.99
**41**	partial cone	244.30	+5.80	1,2,4-alternate	339.96	+4.68	1,2,4,6,8- alternate	443.61	+2.02
**42**	partial cone	244.30	+5.80	1,2,4-alternate	339.97	+4.69	1,3,5,7-alternate	443.67	+12.08
**43**	1,3-alternate	244.31	+5.81	1,2,4-alternate	339.97	+4.69	¾ cone	443.69	+12.10
**44**	partial cone	244.31	+5.81	1,3,5-alternate	339.99	+4.71	1,2,4,6,8- alternate	443.86	+12.27
**45**	1,3-alternate	244.32	+5.82	1,2,4-alternate	340.02	+4.74	1,2,4,6,8- alternate	443.87	+12.28
**46**	partial cone	244.35	+5.85	1,3,5-alternate	340.03	+4.75	1,2,4,6,8- alternate	443.91	+12.32
**47**	1,3-alternate	244.40	+5.90	1,3,5-alternate	340.05	+4.77	1,2,4,6,8- alternate	443.96	+12.37
**48**	partial cone	244.42	+5.92	1,2,4-alternate	340.06	+4.78	1,2,4,6,8- alternate	443.98	+12.39
**49**	partial cone	244.43	+5.93	1,2,4-alternate	340.08	+4.80	1,2,4,6,8- alternate	444.00	+12.41

Supplementary Table 2 - Docking scores of the top 10 docked conformers for 4-sulfonic calix[4]arene, 4-sulfonic calix[6]arene and 4-sulfonic calix[8]arene in AIM2 HIN domain (PDB: 3RN5) using MOE. Related to Figure 4.

MOE					
**4-Sulfonic calix[4]arene**		**4-Sulfonic calix[6]arene**		**4-Sulfonic calix[8]arene**	
**Docking conformation**	**Docking score (Kcal/mol)**	**Docking conformation**	**Docking score (Kcal/mol)**	**Docking conformation**	**Docking score (Kcal/mol)**
1,2-alternate	-6.7	**1,3-alternate**	-8.4	1,2,4,6,8- alternate	-10.2
Cone	-5.8	1,3- alternate	-8.4	**1,2,4,6,8- alternate**	-9.9
1,2-alternate	-5.5	1,3- alternate	-8.3	¾ cone	-9.9
1,2-alternate	-5.5	partial cone	-8.3	1,2,4,6,8- alternate	-9.8
1,2-alternate	-5.5	partial cone	-8.1	1,2,4,6,8- alternate	-9.6
1,2-alternate	-5.5	partial cone	-8.1	¾ cone	-9.5
1,2-alternate	-5.5	1,3,5- alternate	-8.1	1,2-alternate	-9.4
**1,2-alternate**	-5.0	1,2,4- alternate	-8.1	1,2,4,6,8- alternate	-9.4
1,2-alternate	-4.5	1,4- alternate	-7.8	Pleated loop	-9.4
1,2-alternate	-4.4	1,4- alternate	-7.8	1,3,5,7-alternate	-9.3

Supplementary Table 3 - Docking scores of the top 20 docked conformers for 4-sulfonic calix[4]arene, 4-sulfonic calix[6]arene and 4-sulfonic calix[8]arene in AIM2 HIN domain (PDB: 3RN5) using Autodock Vina. Related to Figure 4.

Conformer docking pose	AutoDock Vina																											
	4-Sulfonic calix[4]arene				4-Sulfonic calix[6]arene								4-Sulfonic calix[8]arene															
	Docking score (Kcal/mol)				Docking score (Kcal/mol)								Docking score (Kcal/mol)															
	**1,2-alternate**	**partial cone**	**1,3-alternate**	**cone**	**1,2-alternate**	**partial cone**	**1,4-alternate**	**1,2,4-alternate**	**1,2,3-alternate**	**1,3-alternate**	**1,3,5-alternate**	**cone**	**1,2-alternate**	**1,3,5,7-alternate**	**1,4-alternate**	**1,2,4-alternate**	**1,2,4,6,8-alternate**	**1,5-alternate**	**1,3,5-alternate**	**1,4,5-alternate**	**1,2,3,4-alternate**	**1,3,4,6-alternate**	**1,4,5,6-alternate**	**1,2,5-alternate**	**1,3,4,5-alternate**	**1,2,4,5-alternate**	**¾ cone**	**Pleated loop**
**1**	-16.2	-16.1	-15.3	-16.1	-16.0	-16.2	-15.2	-16.3	-16.1	-15.4	-15.3	-15.8	-13.6	-15.9	-14.9	-15.4	-14.5	-15.6	-15.9	-14.4	-15.7	14.7	-15.7	-13.4	-16.0	-15.7	-14.4	-14.7
**2**	-15.2	-16.1	-15.1	-15.6	-14.6	-16.2	-14.4	-15.2	-15.1	-14.6	-14.6	-15.8	-13.5	-15.3	-14.3	-14.9	-14.2	-15.3	-15.6	-14.0	-14.9	-13.9	-14.9	-13.0	-13.1	-14.9	-13.4	-14.5
**3**	-15.0	-14.3	-14.9	-13.8	-14.3	-16.0	-14.3	-14.8	-14.4	-13.2	-14.4	-15.7	-13.2	-14.9	-14.2	-14.3	-14.1	-13.6	-14.7	-13.7	-14.5	-13.8	-14.2	-12.8	-12.8	-14.5	-13.1	-13.6
**4**	-14.9	-14.2	-14.7	-13.7	-14.2	-14.6	-14.2	-14.8	-14.3	-13.1	-13.4	-15.2	-13.2	-13.9	-14.1	-14.3	-13.9	-13.4	-14.7	-13.6	-14.5	-13.6	-14.1	-12.7	-12.7	-14.1	-13.0	-13.6
**5**	-14.8	-13.5	-14.5	-13.6	-14.1	-14.5	-14.1	-14.7	-14.3	-12.5	-13.3	-15.1	-13.2	-13.8	-13.2	-14.1	-13.8	-13.3	-14.6	-13.6	-14.0	-13.6	-14.0	-12.5	-12.6	-13.9	-12.8	-13.3
**6**	-14.6	-13.2	-14.4	-13.5	-13.7	-13.0	-13.6	-14.2	-14.0	-12.4	-13.1	-14.7	-13.1	-13.2	-13.1	-13.1	-13.4	-12.9	-14.4	-13.4	-13.9	-13.6	-13.9	-12.3	-12.4	-13.7	-12.7	-13.3
**7**	-14.0	-13.2	-14.4	-13.3	-13.1	-13.0	-13.5	-14.1	-13.9	**-12.4**	-13.1	-13.7	-12.3	-13.0	-13.0	-12.8	**-13.3**	-12.5	-14.3	-13.3	-13.8	-13.5	-12.6	-12.3	-12.2	-13.1	-12.7	-13.3
**8**	-13.3	-13.2	-14.4	-13.1	-12.8	-12.9	-13.3	-13.8	-13.9	-12.1	-13.0	-13.3	-12.2	-13.0	-12.9	-12.5	-13.0	-12.5	-13.9	-13.2	-13.5	-13.4	-12.3	-11.7	-11.7	-12.8	-12.6	-13.1
**9**	-13.1	-13.2	-14.0	-12.7	-12.5	-12.6	-13.0	-13.4	-13.9	-12.1	-12.8	-13.3	-12.1	-12.8	-12.9	-12.2	-11.9	-12.2	-13.7	-13.0	-13.3	-13.4	-12.2	-11.7	-11.5	-12.8	-12.5	-12.9
**10**	-13.0	-13.1	-13.9	-12.7	-12.4	-12.5	-12.8	-13.1	-13.6	-12.1	-12.8	-13.2	-11.9	-12.8	-12.6	-12.1	-11.6	-12.2	-13.7	-13.0	-13.3	-13.3	-12.1	-11.6	-11.5	-12.8	-12.4	-12.6
**11**	-12.9	-12.8	-13.7	-12.5	-12.3	-12.4	-12.5	-12.9	-13.5	-12.0	-12.7	-13.1	-11.7	-12.8	-12.5	-12.0	-11.5	-12.2	-12.6	-12.8	-13.0	-13.1	-11.9	-11.6	-11.4	-12.6	-11.9	-12.3
**12**	-12.9	-12.7	-13.3	-12.5	-12.0	-12.2	-12.5	-12.5	-13.3	-11.8	-12.6	-12.5	-11.7	-12.5	-12.5	-11.9	-11.2	-12.2	-12.5	-12.8	-12.6	-13.0	-11.8	-11.1	-11.3	-12.6	-11.6	-11.9
**13**	-12.6	-12.3	-13.2	-12.3	-11.9	-12.0	-12.5	-12.2	-13.3	-11.7	-12.6	-12.4	-11.3	-12.5	-12.5	-11.8	-11.2	-12.1	-12.3	-12.8	-12.3	-12.9	-11.8	-11.1	-11.0	-12.2	-11.5	-11.9
**14**	-12.5	-12.1	-12.5	-12.0	-11.9	-11.9	-12.1	-12.0	-13.2	-11.6	-12.5	-12.0	-11.3	-12.4	-12.2	-11.4	-10.8	-12.0	-12.0	-12.5	-12.1	-12.7	-11.5	-10.9	-10.7	-12.2	-11.4	-11.6
**15**	-12.4	-12.0	-12.2	-11.5	-11.9	-11.8	-12.1	-11.5	-13.2	-11.6	-12.5	-11.9	-11.1	-12.3	-12.0	-11.3	-10.7	-12.0	-11.8	-12.5	-11.8	-12.6	-11.5	-10.9	-10.5	-11.9	-11.3	-11.0
**16**	-12.4	-12.0	-12.1	-11.5	-11.9	-11.7	-12.0	-11.4	-13.2	-11.3	-12.1	-11.8	-11.1	-12.3	-12.0	-11.3	-10.5	-11.9	-11.7	-12.5	-11.6	-12.5	-11.4	-10.8	-10.4	-11.7	-10.9	-10.9
**17**	-12.2	-11.9	-12.0	-11.2	-11.8	-11.7	-11.9	-11.2	-13.0	-11.2	-12.0	-11.7	-10.8	-12.1	-12.0	-11.3	-10.5	-11.8	-11.7	-12.4	-11.3	-12.3	-11.4	-10.7	-10.3	-11.7	-10.9	-10.6
**18**	-12.1	-11.7	-12.0	-11.1	-11.8	-11.3	-11.9	-11.2	-12.8	-11.0	-11.7	-11.7	-10.7	-12.1	-11.8	-11.3	-10.4	-11.4	-11.5	-12.2	-11.2	-12.2	-11.1	-10.7	-10.3	-11.5	-10.9	-10.4
**19**	-11.9	-11.5	-12.0	-11.1	-11.6	-11.3	-11.7	-11.1	-12.4	-11.0	-11.7	-11.4	-10.6	-11.9	-11.7	-11.3	-10.3	-11.3	-11.2	-12.1	-10.7	-12.0	-11.0	-10.7	-10.3	-11.3	-10.8	-10.4
**20**	**-11.7**	-11.4	-11.9	-10.7	-11.5	-11.2	-11.3	-10.9	-12.2	-10.8	-11.6	-11.3	-10.2	-11.8	-11.7	-11.1	-10.3	-11.2	-11.1	-12.1	-10.6	-11.9	-11.0	-10.5	-10.1	-11.2	-10.7	-10.4

Supplementary Figure 7

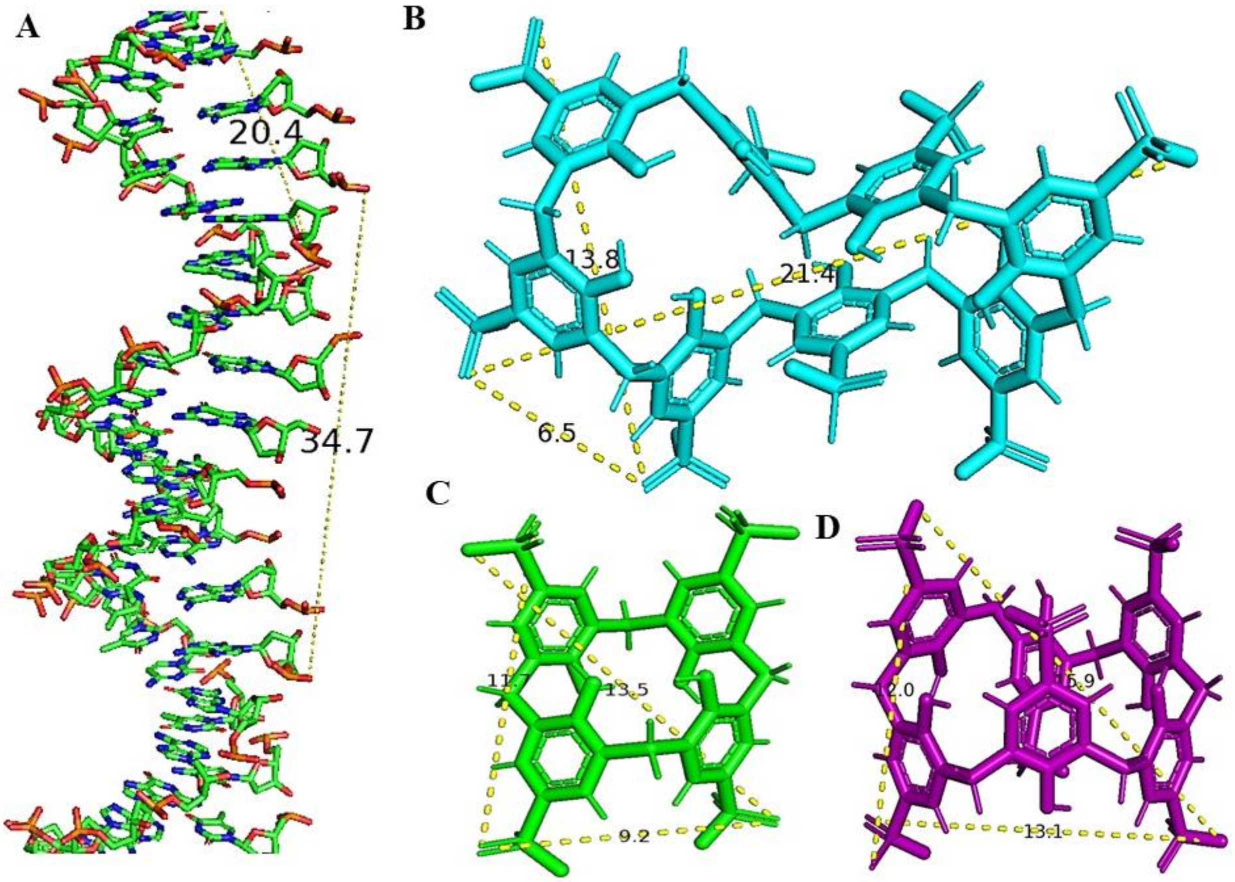

Supplementary Figure 7 - Largest measured O-O distances of dsDNA and 4-sulfonic calixarenes. Related to Figure 4. Yellow dashed lines show largest O-O distances in **(A)** co-crystallized dsDNA in AIM2 for minor groove and major groove regions (PDB: 3RN5), **(B)** 4-sulfonic calix[8]arene, **(C)** 4-sulfonic calix[4]arene, **(D)** 4-sulfonic calix[6]arene. Image created using Pymol.

Supplementary Figure 8

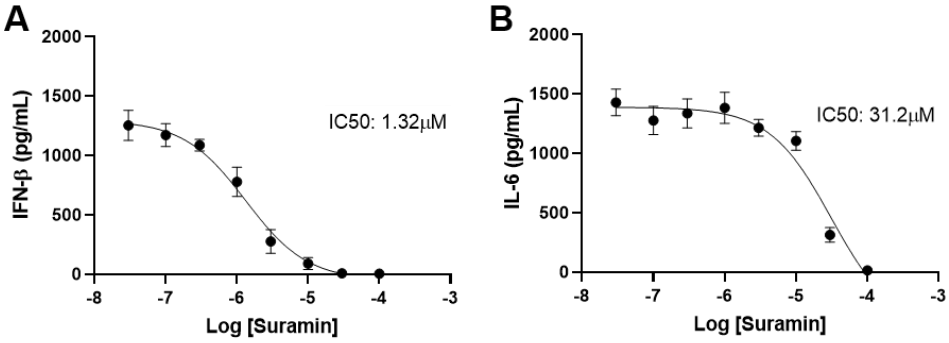

Supplementary Figure 8 – Suramin is an inhibitor of dsDNA-induced IFN-β release and CpG-induced IL-6 release. Related to Figure 6. **(A)** IFN-β release into the supernatants of bone marrow derived macrophages (BMDMs). BMDMs were treated with the indicated concentration of suramin (0.03 – 100 µM) before transfection with poly dA:dT (1 µg mL^{-1}, 6 h) (n=4). **(B)** IL-6 release in the supernatants of BMDMs pre-treated with the indicated concentration of suramin (0.03 – 100 µM) and stimulated with CpG DNA (1 µM, 6 h) (n=4). Concentration-response curves were fitted using a four parameter logistical (4PL) model. Values shown are mean ± the SEM.

Supplementary Figure 9

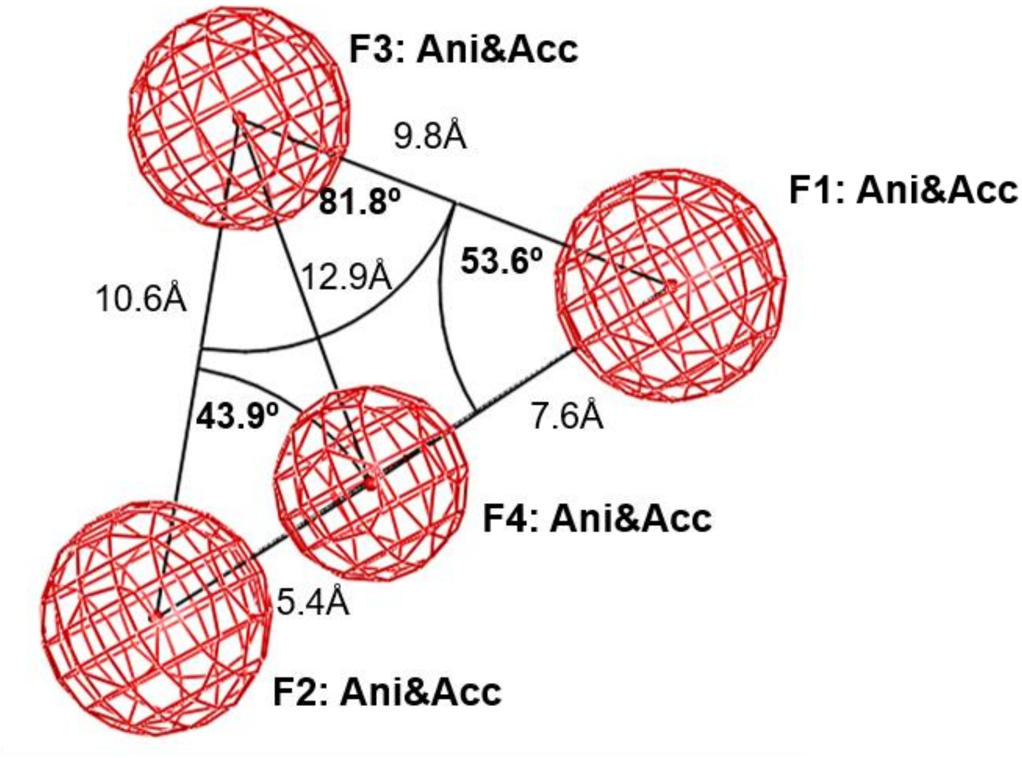

Supplementary Figure 9 - Pharmacophore model of the dsDNA binding site of the HIN domain of AIM2. Related to Figure 6. A pharmacophore model derived from the best binding modes from the docking of 4-sulfonic calixarenes in the AIM2 HIN domain (PDB:3RN5) using Autodock Vina and MOE after overlay of the docked poses. The pharmacophore consists of four ionic interaction sites with radii of 2.1Å, 2.4Å, 2.5Å and 2.5Å (red spheres). Distances and dihedral angles are shown.

Supplementary Table 4 - Ionic interactions and docking scores of the conformers (conf1 & conf2) for suramin in AIM2 (PDB: 3RN5) using MOE and Autodock Vina. Related to Figure 6. Distances from the sulfonate oxygens of suramin to the positively charged amino acid side chains in the HIN domain binding pocket.

	**MOE (Å)**	**AutoDock Vina (Å)**	
**Interacting residue**	**Suramin (conf1)**	**Suramin (conf1)**	**Suramin (conf2)**
**Docking score (Kcal/mol)**	-12.2	-18.9	-22.0
**Lys160**	2.7	2.9	-
**Lys162**	2.9	3.1	-
**Lys163**	2.1	2.9	2.6
**Lys198**	3.2	3.5	3.4
**Lys204**	2.8	2.1	-
**Lys251**	-	-	3.2
**Arg311**	1.7*	2.3*	-
**Lys335**	-	-	-

Supplementary Figure 10

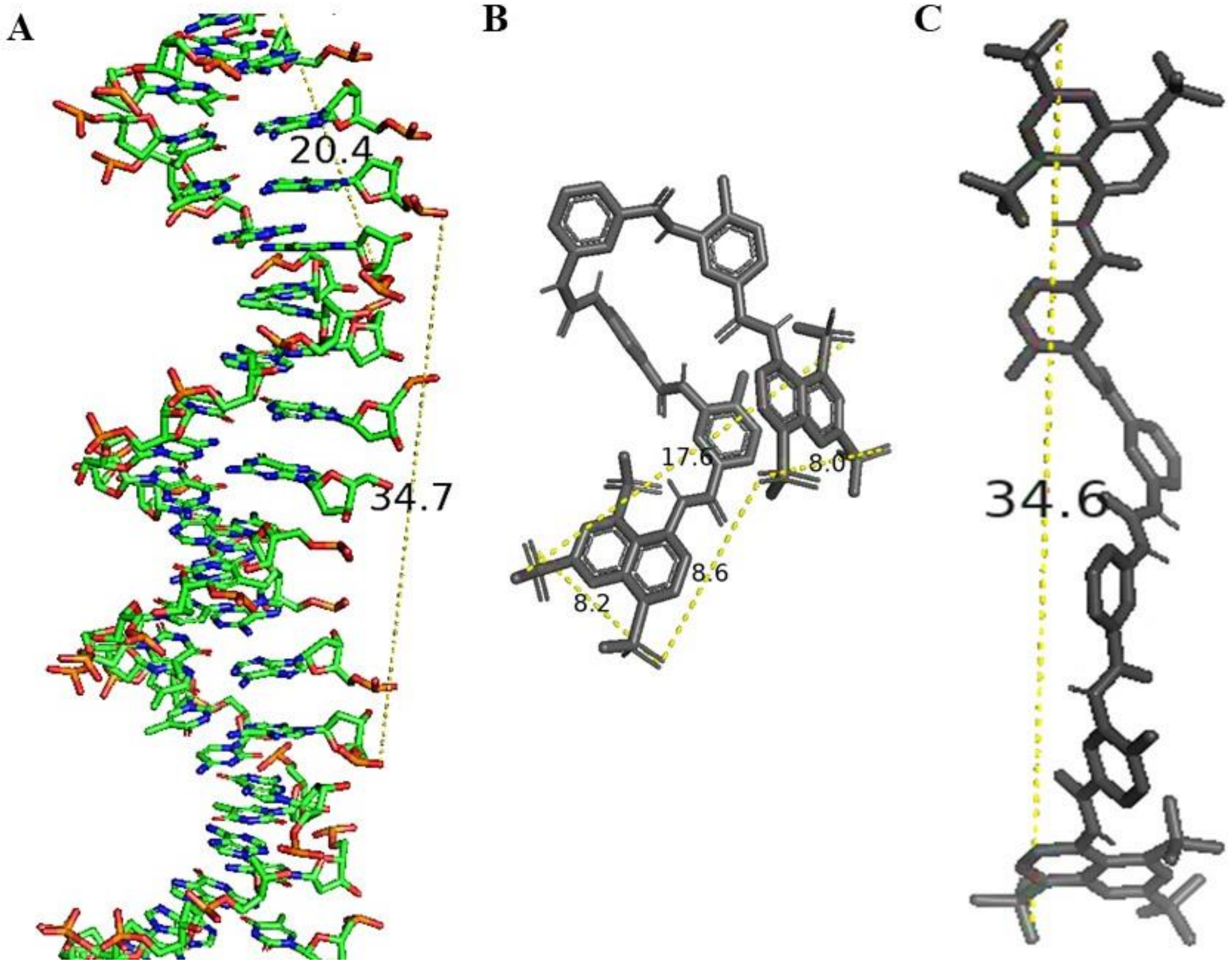

Supplementary Figure 10 - Largest measured O-O distances of dsDNA and suramin. Related to Figure 6. Yellow dashed lines show largest O-O distances in **(A)** co-crystallized dsDNA in AIM2 for minor groove and major groove regions (PDB: 3RN5)**, (B)** preferred docked pose of suramin in the HIN domain (PDB:3RN5), **(C)** extended conformer of suramin for the preferred pose between the 2 HIN domains of AIM2. Image created using Pymol.
